# Ganaxolone improves behavioral deficits in a mouse model of post-traumatic stress disorder

**DOI:** 10.3389/fncel.2014.00256

**Published:** 2014-09-11

**Authors:** Graziano Pinna, Ann M. Rasmusson

**Affiliations:** ^1^The Psychiatric Institute, College of Medicine, University of Illinois at ChicagoChicago, IL, USA; ^2^VA Boston Healthcare System, Women’s Health Science Division of the VA National Center for PTSD, and Boston University School of MedicineBoston, MA, USA

**Keywords:** ganaxolone, allopregnanolone, selective brain steroidogenic stimulants, 5α-reductase type I, PTSD, PTSD therapy, anxiety disorders, GABA_A_ receptor

## Abstract

Allopregnanolone and its equipotent stereoisomer, pregnanolone (together termed ALLO), are neuroactive steroids that positively and allosterically modulate the action of gamma-amino-butyric acid (GABA) at GABA_A_ receptors. Levels of ALLO are reduced in the cerebrospinal fluid of female premenopausal patients with post-traumatic stress disorder (PTSD), a severe, neuropsychiatric condition that affects millions, yet is without a consistently effective therapy. This suggests that restoring downregulated brain ALLO levels in PTSD may be beneficial. ALLO biosynthesis is also decreased in association with the emergence of PTSD-like behaviors in socially isolated (SI) mice. Similar to PTSD patients, SI mice also exhibit changes in the frontocortical and hippocampal expression of GABA_A_ receptor subunits, resulting in resistance to benzodiazepine-mediated sedation and anxiolysis. ALLO acts at a larger spectrum of GABA_A_ receptor subunits than benzodiazepines, and increasing corticolimbic ALLO levels in SI mice by injecting ALLO or stimulating ALLO biosynthesis with a *selective brain steroidogenic stimulant*, such as *S*-norfluoxetine, at doses far below those that block serotonin reuptake, reduces PTSD-like behavior in these mice. This suggests that synthetic analogs of ALLO, such as ganaxolone, may also improve anxiety, aggression, and other PTSD-like behaviors in the SI mouse model. Consistent with this hypothesis, ganaxolone (3.75–30 mg/kg, s.c.) injected 60 min before testing of SI mice, induced a dose-dependent reduction in aggression toward a same-sex intruder and anxiety-like behavior in an elevated plus maze. The EC_50_ dose of ganaxolone used in these tests also normalized exaggerated contextual fear conditioning and, remarkably, enhanced fear extinction retention in SI mice. At these doses, ganaxolone failed to change locomotion in an open field test. Therefore, unlike benzodiazepines, ganaxolone at non-sedating concentrations appears to improve dysfunctional emotional behavior associated with deficits in ALLO in mice and may provide an alternative treatment for PTSD patients with deficits in the synthesis of ALLO. Selective serotonin reuptake inhibitors (SSRIs) are the only medications currently approved by the FDA for treatment of PTSD, although they are ineffective in a substantial proportion of PTSD patients. Hence, an ALLO analog such as ganaxolone may offer a therapeutic GABAergic alternative to SSRIs for the treatment of PTSD or other disorders in which ALLO biosynthesis may be impaired.

## INTRODUCTION

Traumatic life events involving the threat of injury or death, such as combat exposure, sexual assault, witnessing of terroristic attacks, motor vehicle accidents, or involvement in natural disasters may lead to post-traumatic stress disorder (PTSD). PTSD symptoms appear following the traumatic event and fail to extinguish or may worsen over time. PTSD symptoms defined by the Diagnostic and Statistical Manual of Mental Disorders-5 (DSM-5; American Psychiatric Association, 2013) include intrusive memories of the event, recurrent flashbacks and nightmares, emotional and physiological reactions to trauma reminders, difficulty sleeping, trouble concentrating, irritability and aggression, increased startle, hypervigilance, strong negative emotions and beliefs related to the trauma, emotional numbing and avoidance of reminders of the event. An estimated 7–8% of Americans will experience PTSD at some point in their lives, and about 3.6% of U.S. adults aged 18–54 (5.2 million people) will have PTSD during the course of a given year. The prevalence of PTSD in women (10.4%) is about twice that in men (5.0%), representing a relatively small portion of individuals who have experienced at least one traumatic event (60.7% of men and 51.2% of women). However, exposure to certain types of trauma, such as sexual assault and combat, is associated with a substantially higher (15–30%) risk for PTSD. PTSD is also associated with increased rates of other psychiatric and medical comorbidities including depression, anxiety disorders, traumatic brain injury, chronic pain, cardiovascular disorders, metabolic syndrome, and substance abuse, particularly tobacco and alcohol dependence (Rasmusson et al., 2010; Carlson et al., 2011; Friedman et al., 2014; Rasmusson and Shalev, 2014; Scioli-Salter et al., 2014).

Notwithstanding the prevalence of this debilitating psychiatric disorder in the general population, the only FDA-approved drugs for the treatment of PTSD are the selective serotonin reuptake inhibitors (SSRIs; Brady et al., 2000; Davidson et al., 2001; Marshall et al., 2001; Tucker et al., 2001). The response rate to these drugs, however, is relatively small, and some studies have shown that male combat veterans, in particular, may be resistant to their therapeutic effects, although ethnic differences may play a role in veteran response rates (Hertzberg et al., 2000; Zohar et al., 2002; Friedman et al., 2007; Panahi et al., 2011). The search for neurobiological biomarkers for PTSD is therefore a current focus of investigation in the hope that a better understanding of individually variable neurobiological risk factors for PTSD will spur development of more specific and individually effective therapies.

Stress-induced alterations in the composition of GABA_A_/benzodiazepine receptor complexes are involved in the lack of response to classical benzodiazepine ligands as well as in the production of dysfunctional behaviors following stress or traumatic events, as documented in both preclinical and clinical studies. In postmortem studies, alterations in GABA_A_ receptor binding and receptor subunit composition, as well as in in GABA synthesis and transport are associated with anxiety disorders and depression in humans (Vaiva et al., 2004; Geuze et al., 2008). In studies of patients with PTSD, GABA levels are reduced (Kugaya et al., 2003), as are GABA_A_/benzodiazepine receptor binding (Bremner et al., 2000). Similarly, in rodents, chronic stress and fear conditioning have been shown to diminish GABA-mediated neurotransmission within the amygdala (Martijena et al., 2002), by decreasing expression of genes for GABA synthesizing enzymes, decreasing NE α_1_-stimulated GABA release from interneurons within the basolateral nucleus of the amygdala (BLA; Braga et al., 2004), downregulating gephyrin, a protein that anchors synaptic GABA_A_ receptors, and downregulating synaptic GABA_A_ receptors themselves (Chhatwal et al., 2005; Heldt and Ressler, 2007). Together, these studies suggest why benzodiazepines have not been found to be beneficial in treating the core symptoms of PTSD (Geuze et al., 2008). In addition, recent work shows that reductions in GABA synthesis by knockdown of GAD67 in the amygdala, as well as specific knockdown of the GABA_A_ receptor α1 subunit that confers benzodiazepine sensitivity on corticotropin releasing factor (CRF) neurons, disrupts extinction (Gafford et al., 2012; Heldt et al., 2012).

Levels of neurosteroids that positively and allosterically modulate GABA action at GABA_A_ receptors (Puia et al., 1990, 1991; Belelli and Lambert, 2005) also have been found to be low in PTSD patients (Rasmusson et al., 2006). In premenopausal women, cerebrospinal fluid (CSF) levels of ALLO and its equipotent stereoisomer pregnanolone (together termed ALLO) were 40% of the levels seen in healthy subjects and were inversely correlated with PTSD re-experiencing and comorbid depressive symptoms (Rasmusson et al., 2006). In fact, levels were lowest in those PTSD patients with current comorbid depression. In addition, the ratio of ALLO to its steroid precursor, 5α-dihydroprogesterone (5α-DHP), was decreased among the PTSD patients, suggesting dysfunction of the enzymes involved in ALLO synthesis (Rasmusson et al., 2006). Similarly, among recently deployed male veterans, the ratio of ALLO to progesterone, the precursor for 5α-DHP, was lowest in those veterans with the most severe PTSD and depression symptoms (Kilts et al., 2010).

Although neurosteroids such as ALLO have activity at all subtypes of GABA_A_ receptors, they have highest affinity for a benzodiazepine-resistant subset of extrasynaptic GABA_A_ receptors composed of α_4_ and δ subunit combinations or α_6_, γ, and β subunit combinations (Lambert et al., 2003; Belelli and Lambert, 2005). These extrasynaptic receptors are activated by concentrations of GABA lower than that required for activation of synaptically located GABA_A_ receptors. As a consequence, extrasynaptic GABA_A_ receptors are thought to maintain a tonic inhibitory conductance that modulates gain in neuronal output during periods of increased input (Mitchell and Silver, 2003; Semyanov et al., 2003, 2004; Mody and Pearce, 2004; Sun et al., 2004), as occurs during stress. Of note, α_4_, δ, and α_6_ GABA_A_ receptor subunits increase under conditions in which ALLO levels are decreased (Smith et al., 1998; Follesa et al., 2001; Gulinello et al., 2002; Sundstrom-Poromaa et al., 2002; Pinna et al., 2006b). In hippocampus (at least, as other areas have not yet been studied), extrasynaptic GABA_A_ receptors also appear to be reciprocally upregulated when synaptic GABA_A_ receptors are downregulated. This suggests that after fear conditioning when synaptic GABA_A_ receptors are downregulated in the amygdala, maintenance of adequate GABA tone in the amygdala may depend on positive modulation of extrasynaptic GABA_A_ receptors by neurosteroids, such as ALLO, that are synthesized and released locally or that enter the brain after release from the adrenal gland. Thus, pharmacological interventions aimed at normalizing brain ALLO levels in PTSD patients with deficiencies in ALLO synthesis, might be expected to restore GABAergic neurotransmission and enhance recovery from PTSD.

We previously sought to investigate this hypothesis in mice subjected to four weeks of social isolation, which results in a 70% reduction in ALLO and 5α-DHP biosynthesis (Matsumoto et al., 1999; Dong et al., 2001). Importantly, the largest decrease of ALLO induced by social isolation was found in the amygdala and hippocampus, followed by the olfactory bulb and frontal cortex (Pibiri et al., 2008). ALLO levels failed to change in the cerebellum and striatum (Pibiri et al., 2008). *In situ* immunohistochemical studies further demonstrated that 5α-reductase conversion of 5α-DHP to ALLO, the rate-limiting enzymatic step in ALLO biosynthesis, was specifically decreased in cortical pyramidal neurons of layers V–VI, hippocampal CA3 pyramidal neurons, glutamatergic granular cells of the dentate gyrus, and pyramidal-like neurons of the basolateral amygdala (Agís-Balboa et al., 2007). Notably, brain interconnections arising from these corticolimbic areas play a primary role in the regulation of emotional behavior, including fear responses, as demonstrated by both human and basic research studies (Myers and Davis, 2007). Accordingly, in SI mice, downregulation of ALLO biosynthesis was associated with the emergence of neurobehavioral dysfunction including anxiety-like behavior and aggression towards same-sex intruders (Matsumoto et al., 1999; Pinna et al., 2003, 2006a, 2008; Pibiri et al., 2008). Furthermore, SI mice exposed in a novel environment (i.e., the context) to the administration of acoustic tones preceding unconditioned footshock stimuli, exhibited exaggerated conditioned contextual fear response and impaired fear extinction (Pibiri et al., 2008; Pinna et al., 2008). Thus, protracted social isolation combined with fear-conditioning could be a suitable mouse model to study emotional behaviors and neurochemical alterations related to PTSD (Pibiri et al., 2008; Pinna, 2010).

Similar to PTSD patients, SI mice also show resistance to classical benzodiazepine ligands such as diazepam and zolpidem in association with changes in mRNA and protein expression for several GABA_A_ receptor subunits in the frontal cortex and hippocampus (Pinna et al., 2006b; Nin Schuler et al., 2011). Expression of GABA_A_ receptor subunits α1, α2, and γ2 were reduced by approximately 50%, whereas the mRNAs encoding α5 and α4 subunits, which confer increased sensitivity to neuroactive steroids such as ALLO, were increased by approximately 130% compared to levels in group-housed control mice (Pinna et al., 2006b). In the SI mice, the systemic administration of ALLO or infusion of ALLO directly into the basolateral amygdala had a strong anti-aggressive effect (Nelson and Pinna, 2011). These results were replicated by the administration of *S*-norfluoxetine at doses that failed to have serotonergic effects but potently increased ALLO biosynthesis in target corticolimbic areas, including the hippocampus, basolateral amygdala, and frontal cortex (Pinna et al., 2006a; Nelson and Pinna, 2011).

The present translational study was undertaken to evaluate whether ganaxolone (3α-hydroxy-3β-methyl-5α-pregnan-20-one), a 3β-methylated synthetic analog of allopregnanolone (ALLO) that cannot be converted back into its progesterone precursors, has a similar capacity to improve anxiety and PTSD-like behaviors manifested by SI mice, including increased aggression and exaggerated contextual fear responses. Ganaxolone has shown efficacy as an anticonvulsant in a number of animal models (e.g., Reddy and Rogawski, 2010), and is currently being investigated for the treatment of refractory epilepsy (Bialer et al., 2013) and PTSD in human clinical trials^1^.

## MATERIALS AND METHODS

### SUBJECTS

**FIGURE 1:**
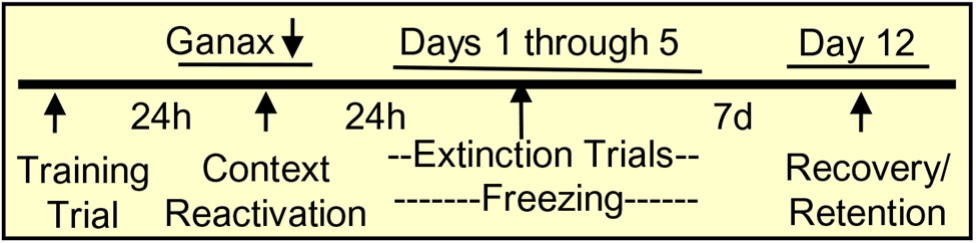
**Contextual fear conditioning protocol.** Mice were trained in the conditioning chamber by tone plus footshock, which was repeated three times every 2 min. The total time in the conditioning chamber was 8 min. To induce retrieval/reactivation of the training memory, mice were placed in the conditioning chamber for 5 min and immediately after the reactivation session, they received a single injection of vehicle or ganaxolone. For the extinction trial (5 days), mice were placed in the chamber for 5 min without footshock, and freezing was measured as an indication of contextual fear. After an interval of 7 days (day 12), mice were reexposed to the chamber without footshock and freezing was measured as an indicator of the spontaneous reinstatement of contextual fear, or inversely, as extinction retention.

Adult male Swiss–Webster mice (Harlan Breeders, Indianapolis), 18–20 g body weight, were maintained under a 12-h dark/light cycle and provided food and water *ad libitum* in a vivarium with temperature and humidity kept near 24°C and 65%, respectively. SI mice were housed individually in a 24 × 17 × 12 cm cage for 3–4 weeks, while group-housed control mice were housed in groups of 5. Ganaxolone was obtained from Marinus Pharmaceuticals, Inc^2^. Ganaxolone, pregnanolone, or vehicle (corn oil) in a volume of 100 μl/10 g was injected subcutaneously (s.c.) 60 min before behavioral tests of locomotor activity, anxiety-like behavior, and aggressive behavior toward an intruder in the home cage. In a study of ganaxolone effects on fear extinction and retention, ganaxolone or vehicle was administered just once, immediately after the first session in which the mice were reexposed to the context in which fear conditioning was performed the day before (i.e., subsequent to the first reactivation or extinction session; **Figure 1**). All experimental protocols were approved by the Office of Animal Care and Institutional Biosafety Committee and the Office of the Vice Chancellor for Research of the University of Illinois at Chicago.

## BEHAVIORAL TESTING

### ELEVATED PLUS MAZE

Behavioral testing was performed between 10.00 and 14.00 h in a light- and sound-controlled room using an elevated plus-shaped maze constructed of black acrylic and elevated 50 cm above the floor (Uz et al., 2004). In this test, mice chose between entering the two relatively anxiogenic 45 × 10 cm open arms and the two relatively safe 45 × 10 × 12 cm closed arms that extended from a 10 × 10 cm central platform. Mice were initially placed facing the closed arm. Entry onto an arm with less than four legs was counted as a crossing. An arm entry was scored when all four legs were within the arm. Behavior in the maze was recorded and scored for 10 min, 60 min after the single s.c., injection of ganaxolone (3.75–30 mg/kg) or vehicle (corn oil). Time spent on the open arm and the number of open arm crossings, closed arm crossings, and closed arm entries were analyzed. After each test, the maze was wiped with ethanol/water (50% v/v).

### RESIDENT–INTRUDER TEST

To test aggression, a male intruder mouse of the same strain as the resident mouse, was placed in a resident home cage (24 × 17 × 12) and resident–intruder interactions were videotaped for 10 min. Aggressive behavior of SI mice was characterized by an initial pattern of exploratory activity around the intruder, followed by rearing and tail rattle, accompanied within a few seconds by wrestling and/or a violent biting attack. The total duration of wrestling and attack behavior during the 10 min observation period was measured as previously described (Pinna et al., 2003, 2005), 60 min after administration of a single dose of ganaxolone (3.75–30 mg/kg, s.c.). To establish whether ganaxolone is superior to ALLO in decreasing aggressiveness of SI mice, an EC_50_ dose of ganaxolone (10 mg/kg, s.c.) was used in a comparison experiment with the same dose of pregnanolone (10 mg/kg, s.c.). Behavioral testing was performed between 10.00 and 14.00 h.

### CONTEXTUAL FEAR CONDITIONING

#### Apparatus

The conditioning and extinction chamber (25 cm wide, 18 cm high, and 21 cm deep) had a cage floor made of stainless steel rods connected to an electric shock generator (San Diego Instrument, Inc., San Diego, CA). It was surrounded by a frame that emitted 16 infrared photo beams. A computer controlled the delivery of electric footshocks and recorded beam interruptions and latencies to beam interruptions (freezing time).

#### Conditioning trial

The group-housed and SI mice were placed in the chamber and allowed to explore for 2 min before exposure to a 30 s, 85 DB acoustic tone (conditioned stimulus, CS) that co-terminated with a 2 s, 0.5 mA electric footshock (unconditioned stimulus, US). The tone plus footshock was repeated three times randomly within each subsequent 2 min epoch. One minute after the last tone-footshock delivery, mice were returned to their home cages. The total time in the conditioning chamber was 8 min.

#### Reactivation

Mice were returned to the chamber 24 h later for 5 min without footshock presentation to induce retrieval/reactivation of the training memory. Immediately after the reactivation session, each mouse received a single s.c., injection of vehicle or EC_50_ dose of ganaxolone (as established in the previous tests of aggression).

#### Contextual fear

Twenty-four hours after the reactivation/first extinction trial, the mice were placed in the chamber for 5 min without footshock, and freezing was measured as an indication of contextual fear.

#### Extinction and extinction retention

Mice were placed in the chamber for the next 5 days in a row starting 24 h after the reactivation session. After a subsequent interval of 7 days (day 12), mice were reexposed to the chamber without footshock and freezing was measured as an indicator of the spontaneous reinstatement of contextual fear, or inversely as extinction retention (**Figure 1**). Freezing was defined as the absence of movement except respiration while the mice remained in a crouched posture (Pibiri et al., 2008).

### MEASUREMENT OF EXPLORATORY ACTIVITY IN A NOVEL CAGE

A computerized AccuScan 12 Animal Activity Monitoring System (Columbus Instruments, Columbus, OH, USA) assisted by VERSAMAX software (AccuScan Instruments, Columbus, OH, USA) was used to quantify locomotor activity (Pinna et al., 1997, 2006b). Each activity cage consisted of a 20 × 20 × 20 cm Perspex box surrounded by horizontal and vertical infrared sensor beams. Horizontal sensors beam interruptions were taken as a measure of horizontal activity, whereas vertical sensor beam interruptions counted as rearing activity. Activity was recorded from group-housed and SI mice between 13.00 and 15.00 h for 15 min beginning 60 min after a single injection of vehicle or various doses of ganaxolone (3.75–30 mg/kg, s.c.).

### STATISTICAL ANALYSES

Results are presented as means ± SEMs unless otherwise indicated. Comparisons between the control group and each of the treatment groups were performed using one-way ANOVA followed by LSD’s test or repeated measures ANOVA followed by a Greenhouse–Geisser correction. Significance was set at *P* < 0.05. Ganaxolone EC_50_ values were calculated from dose–response curves analyzed by the “quantal dose–response: probits test” using the computer program of Tallarida and Murray equipped with a statistical package. Statistical comparisons among the different EC_50_s were performed with the “cohort package software^3^.”

## RESULTS

### DOSE-DEPENDENT GANAXOLONE EFFECTS ON AGGRESSIVE BEHAVIOR IN SI MICE

**FIGURE 2:**
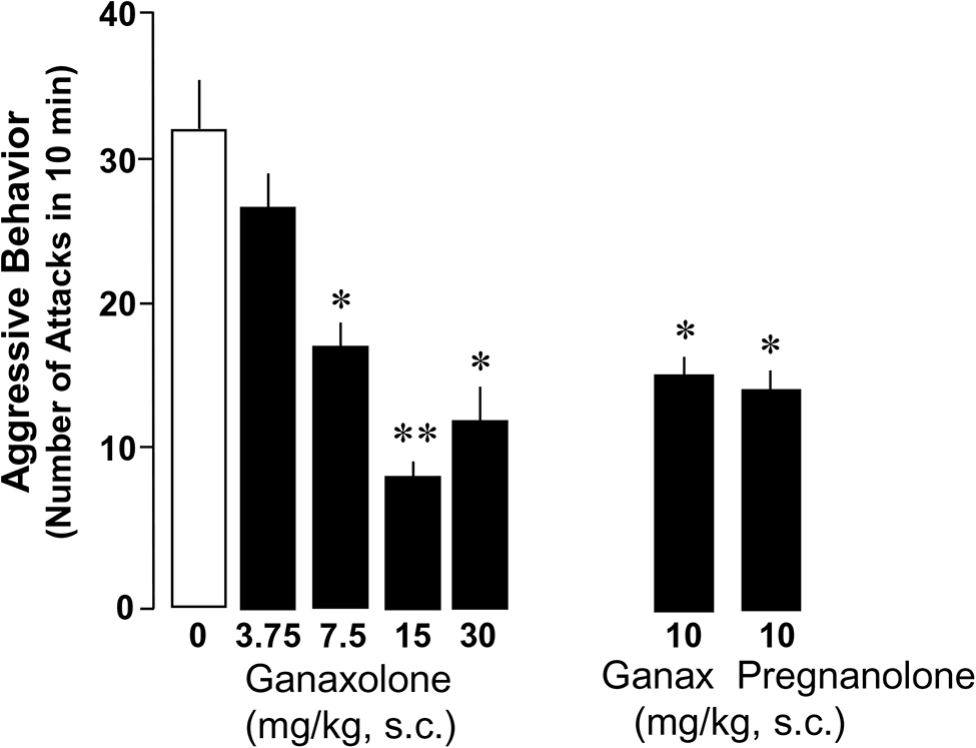
**Ganaxolone dose-dependently decreases social isolation-induced aggression of resident mice toward a same-sex intruder.** Dose–response curve resulted in an EC_50_ dose of 10 mg/kg of ganaxolone. Equimolar doses of ganaxolone and the GABA_A_ receptor active, ALLO isoform, pregnanolone were equipotent in decreasing aggressive behavior in SI mice. Data represent the mean ± SEM of 8–10 SI mice. *P < 0.01; **P< 0.001, when compared with vehicle-treated (0) mice.

Administration of ganaxolone (3.75–30 mg/kg, s.c.) resulted in a dose-dependent decrease of aggressive behavior directed by SI resident mice toward same-sex intruders (**Figure 2**). There was a highly significant main effect of ganaxolone treatment on aggressive behavior (*F*_4,36_ = 6.89, *P* < 0.001). The dose of 30 mg/kg was not more efficacious than the 15 mg/kg dose in decreasing aggression. Equimolar doses of ganaxolone and pregnanolone were equipotent in ameliorating the social isolation-induced aggression. The analyses of the dose–response curve resulted in an EC_50_ of 9.7 mg/kg of ganaxolone, which was the dose used in the evaluation of the contextual fear conditioning response experiments.

### ANXIETY-LIKE BEHAVIOR IN GROUP-HOUSED AND SI MICE TREATED WITH GANAXOLONE

**FIGURE 3:**
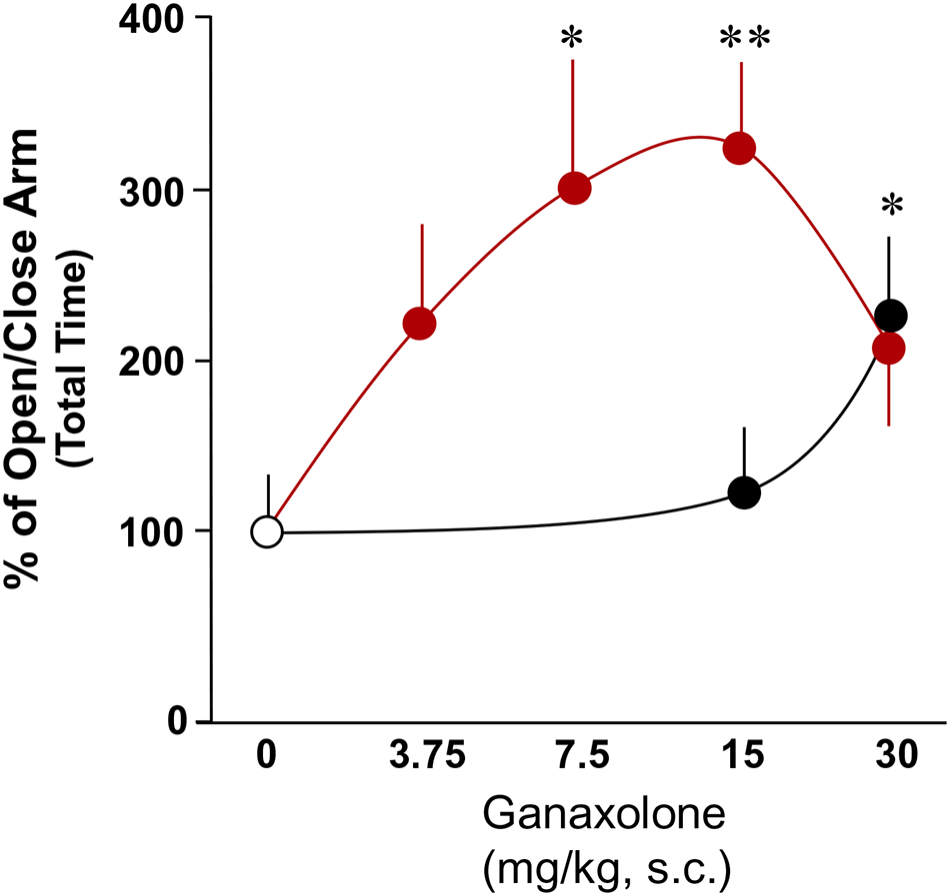
**The effects of ganaxolone on anxiety-like behavior in SI mice (red circles) results in a bell shaped dose–response curve, which is shifted to the right in group-housed mice (black circles).** Ganaxolone in the dose range of 3.75–30 mg/kg improves anxiety-like behavior of SI mice exposed to an elevated plus maze and assessed by open to close arm total time, and improved anxiety-like behavior of group-housed mice at the high dose of 30 mg/kg, s.c. Data represent the mean ± SEM of eight to fourteen mice. *P < 0.05; **P < 0.01 when compared with vehicle-treated (0) mice.

**FIGURE 4:**
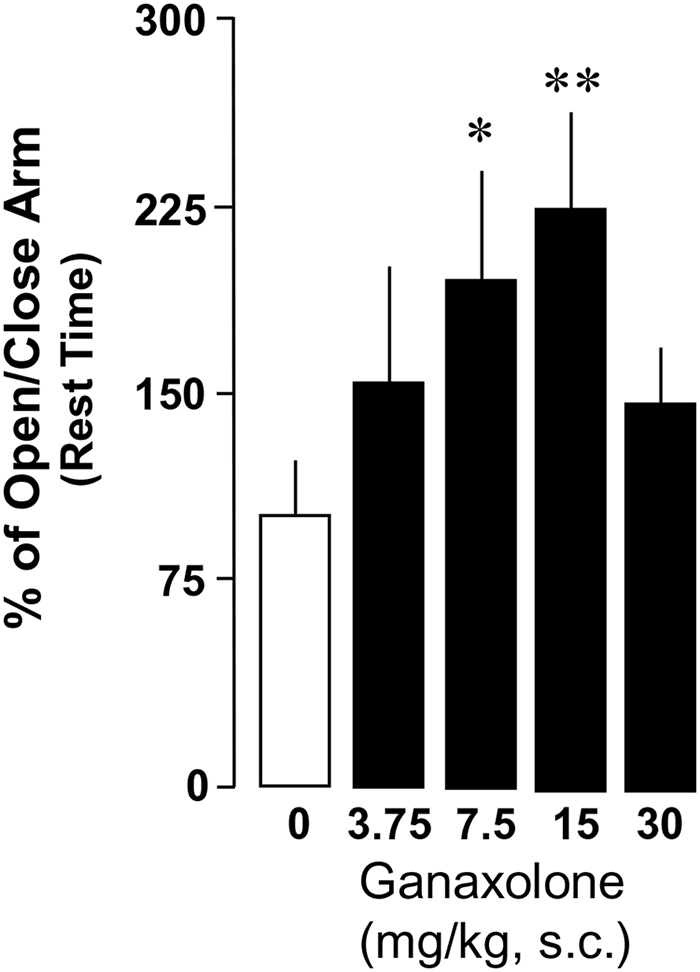
**Ganaxolone dose-dependently decreases social isolation-induced anxiety-like behavior of mice exposed to an elevated plus maze, determined by the ratios of open arm to closed arm rest time.** Data represent the mean ± SEM of 8–14 mice. *P < 0.05; **P < 0.01 when compared with vehicle-treated (0) mice.

This study confirmed findings of previous experiments demonstrating increased anxiety-like behavior in a plus maze in SI mice compared with group-housed mice (Pinna et al., 2006a; Nin Schuler et al., 2011). There was a significant main effect of ganaxolone treatment within SI mice and a dose-dependent effect of ganaxolone treatment on several anxiety-like measures (ratio of open to closed arm total time: *F*_4,41_ = 2.80, *P* = 0.038; ratio of open to closed arm rest time *F*_4,41_ = 2.66, *P* = 0.04; **Figures 3** and **4**). The lowest dose of ganaxolone (3.75 mg/kg) only showed a trend towards improvement of anxiety-like behavior expressed as the ratio of open arm to closed arm total time (*P* = 0.08; **Figure 3**). Ganaxolone treatment at the 7.5 mg/kg dose significantly increased the ratios of open arm to closed arm rest time as well as total time spent in the open arms (*P* = 0.02 and *P* = 0.01, respectively). The most effective 15 mg/kg dose of ganaxolone induced anxiolytic effects as determined by the ratios of open arm to closed arm rest time and total time (*P* = 0.007 for both measures). The dose of 30 mg/kg did not elicit an improvement of social isolation-induced anxiety-like behavior (**Figures 3** and **4**).

In group-housed mice, there was a significant main effect of ganaxolone treatment (ratio of open to closed arm total time: *F*_2,22_ = 4.46, *P* = 0.027). Ganaxolone at a dose of 15 mg/kg, s.c., did not affect anxiety-like measures. The highest 30 mg/kg ganaxolone dose did, however, induce an anxiolytic effect as mice treated with this dose showed an increase in the ratio of open arm to closed arm total time (*P* = 0.04; **Figure 3**) and in the ratio of open arm to closed arm distance traveled (*P* = 0.027; not shown).

### CONTEXTUAL FEAR RESPONSES IN SI MICE THAT RECEIVED AN EC_50_ DOSE OF GANAXOLONE

**FIGURE 5:**
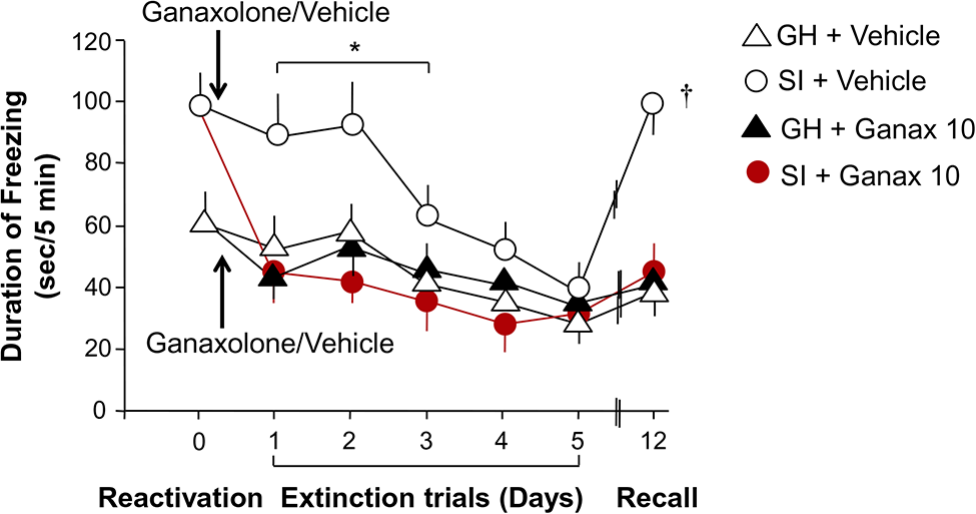
**Ganaxolone facilitates fear extinction and blocks contextual fear reconsolidation.** SI mice (empty circles) exhibit increased freezing and reduced extinction compared to group-housed mice over a period of five extinction trials. Ganaxolone treatment administered immediately after a reactivation session (black arrow) compared to vehicle treatment resulted in less freezing in the SI mice (red circles). Ganaxolone did not affect freezing time in the group-housed mice (black triangle). Importantly, ganaxolone prevented the spontaneous reemergence of contextual fear responses after the passage of time – or from another perspective, enhanced extinction retention in SI mice. Data represent the mean ± SEM of 10–12 mice. *P = 0.035 when compared to SI + Ganaxolone; **^†^***P* = 0.025 when compared to SI + ganaxolone on recall (day 12).

SI mice compared to group-housed mice exposed to contextual fear conditioning exhibited increased freezing and reduced extinction over a period of five extinction trials (**Figure 5**). Repeated-measures ANOVA with a Greenhouse–Geisser correction showed a significant group by drug treatment by extinction session interaction for freezing across extinction sessions day 1–3, the time interval over which extinction continued to decline (*F*_1.995,43.885_ = 3.618; *P* < 0.035). *Post hoc* testing revealed that ganaxolone treatment compared to vehicle treatment resulted in less freezing in the SI mice. Ganaxolone did not affect freezing time in the group-housed mice. Importantly, the single EC_50_ dose (10 mg/kg) of ganaxolone administered after the first fear reactivation/extinction session prevented the spontaneous reemergence of contextual fear responses after the passage of time—or from another perspective, enhanced extinction retention (*T*_1,23_ = 5.809, *P* = 0.025; **Figure 5**).

### EFFECTS OF GANAXOLONE ON EXPLORATORY ACTIVITY IN SI AND GROUP-HOUSED MICE

**FIGURE 6:**
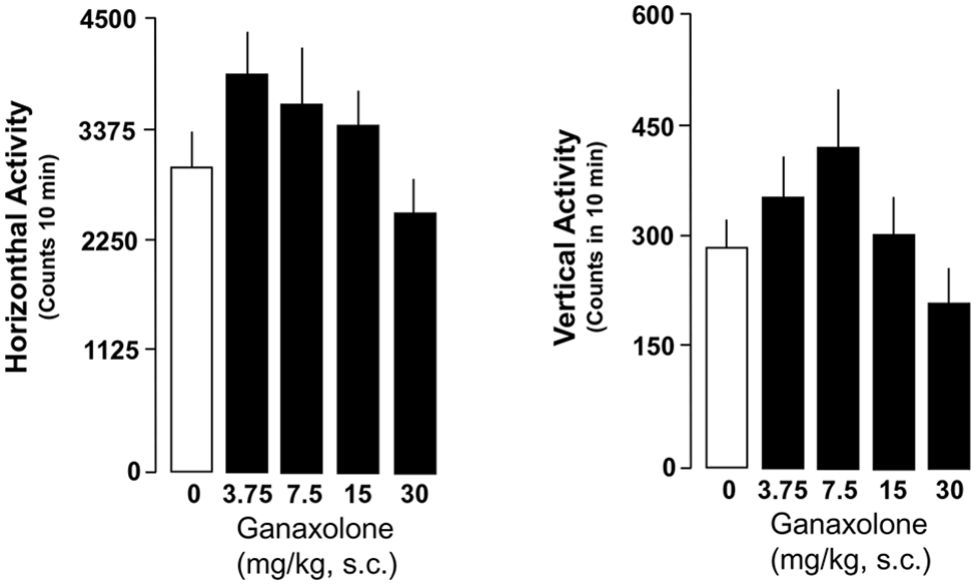
**Ganaxolone did not alter exploratory activity determined as means of horizontal and vertical activity in SI mice even at the highest dose (30 mg/kg) tested**. The lowest doses of ganaxolone (3.75 and 7.5 mg/kg, s.c.) exhibit a trend to increase both horizontal and vertical locomotor activity in SI mice. Data represent the mean ± SEM of six to eight SI mice.

**FIGURE 7:**
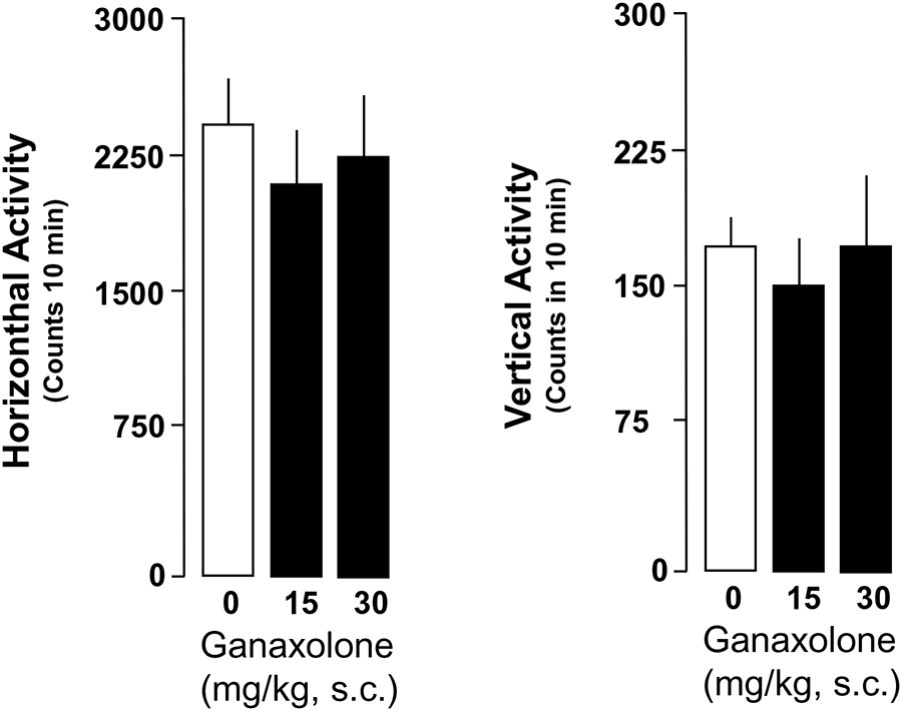
**Ganaxolone fails to change locomotor measures assessed as horizontal and vertical activity in group-housed mice**. Data represent the mean ± SEM of six group-housed mice.

Ganaxolone did not reduce exploratory activity in either SI or group-housed mice, even at the highest dose (30 mg/kg) tested. There was a trend for the lowest doses of ganaxolone (3.75 and 7.5 mg/kg) to stimulate both horizontal and vertical locomotor activity in SI mice (**Figures 6** and **7**).

## DISCUSSION

This study assessed the effects of a synthetic ALLO analog, the neuroactive steroid ganaxolone, on anxiety-like behavior, aggression, and contextual fear conditioning and extinction, as well as locomotor activity in male mice. Importantly, ganaxolone administered s.c. at 3.75–30 mg/kg did not impair exploratory activity as assessed by characterization of horizontal and vertical locomotion patterns. Ganaxolone did, however, show a strong anxiolytic effect in mice tested in the elevated plus maze, with lower doses effective in SI mice with deficits in ALLO, and higher doses effective in group-housed mice with normal ALLO levels. Ganaxolone also dose-dependently decreased aggression in SI mice to a same-sex intruder at doses comparable to ALLO doses with comparable effects. Most intriguingly, an EC_50_ dose (10 mg/kg, s.c.) of ganaxolone, given immediately after reactivation of contextual fear 1 day after fear conditioning, substantially diminished contextual fear on subsequent test days in SI mice. In addition, it blocked the spontaneous reemergence of contextual fear a week after extinction was complete – or from another perspective, corrected deficits in extinction retention exhibited by SI mice. Of note, such deficits in extinction retention have been observed in studies of PTSD in humans (e.g., Milad et al., 2008), thus reinforcing the idea that deficiencies in GABAergic neurotransmission associated with deficient ALLO biosynthesis constitute a vulnerability to the development of PTSD-like behaviors in response to threat, modeled in this study by exposure to footshock in a Pavlovian fear conditioning paradigm.

These data are in agreement with previous reports demonstrating strong anxiolytic effects of ganaxolone at 10 mg/kg i.p. in rats (Kudagi et al., 2012) and wild-type or GABA_A_ receptor delta subunit knockout mice (Mihalek et al., 1999). These results thus suggest that ganaxolone may be useful in clinical practice for a subpopulation of patients in whom anxiety or PTSD symptoms are related to deficient ALLO biosynthesis. It is possible that ganaxolone also may find application in other disorders characterized by a downregulation of brain ALLO levels, including depression (Uzunova et al., 1998; Agis-Balboa et al., 2014).

### CURRENT PTSD TREATMENT OPTIONS

Currently, there is no specific pharmacological treatment for PTSD. The only FDA approved medications for the management of this debilitating disorder are the serotonin selective reuptake inhibitors (SSRIs), paroxetine and sertraline. Although SSRIs improve symptoms of PTSD in some patients, meta-analyses have demonstrated that response rates rarely exceed 60% and that only 20–30% of patients achieve a full remission of symptoms (Westenberg, 1996; Walderhaug et al., 2010; Ipser and Stein, 2012). Venlafaxine, a serotonin–norepinephrine reuptake inhibitor (SNRI) was shown to induce a positive clinical response in 78% of PTSD patients (Davidson et al., 2006). However, only 40% of patients who completed the treatment achieved PTSD remission and the drug was not effective for PTSD hyperarousal symptoms (Davidson et al., 2006).

The finding that low non-serotonergic doses of fluoxetine and congeners increase ALLO levels as their primary mechanism of action, suggests that SSRIs acting as *selective brain steroidogenic stimulants* (SBSSs; Pinna et al., 2003, 2006a; Pinna, 2014) may thereby improve psychiatric symptoms and be of use in PTSD (reviewed in Pinna and Rasmusson, 2012). However, the high rate of resistance to current medications of this class suggests that deficits in the activity of enzymes involved in ALLO synthesis may not be amenable to correction by SSRIs in PTSD—and/or that the pathophysiology of PTSD is more complex and/or varies among individuals. The study by Rasmusson et al. (2006), suggested that 3α-hydroxysteroid dehydrogenase (3α-HSD) activity is downregulated in premenopausal women with PTSD. Work by Gillespie et al. (2013), on the other hand, showed that a polymorphism of the 5α-reductase type I gene predicted an increase in the risk for PTSD in men. This suggests the possibility that the specific enzyme site responsible for ALLO deficiency in PTSD may differ between men and women, as supported by the work of Pinna et al. (2008) showing that social isolation downregulates corticolimbic ALLO levels in male rodents at 5α-reductase, but not female rodents, unless the females are oophorectomized and replaced with testosterone (Pinna et al., 2005). Hence, it is possible that SSRIs and SNRIs currently in use for the treatment of PTSD do not adequately enhance gene expression or enzymatic function at these sites in individuals resistant to their therapeutic effects. Therefore, an alternative strategy might be to directly activate GABA_A_ receptors with an ALLO analog such as ganaxolone (Gulinello et al., 2003; Kaminski et al., 2004; Pinna, 2014).

### OTHER NEUROSTEROIDOGENIC DRUGS FOR THE POTENTIAL TREATMENT OF PTSD-LIKE SYMPTOMS

There are several other neurosteroidogenic biomarkers with the potential to serve as targets for the next generation of anxiolytic, antidepressant, or PTSD relevant drugs. One of the best studied is the 18 kDa translocase protein (TSPO; Papadopoulos et al., 2006; Rupprecht et al., 2009, 2010; Schüle et al., 2011, 2014), formally known as the peripheral benzodiazepine receptor (Costa and Guidotti, 1991; Costa et al., 1994). TSPO regulates the availability of neurosteroids in the brain by facilitating access of cholesterol to the inner mitochondrial membrane and its subsequent conversion to pregnenolone by the rate-limiting step enzyme, P450scc, located within the inner mitochondrial membrane (Papadopoulos et al., 2006; Rupprecht et al., 2009, 2010). TSPO agents have been shown to potently increase ALLO levels in brain regions that regulate emotional behavior, such as the hippocampus and cortex, and to induce anxiolytic effects (Kita et al., 2004). Several TSPO ligands have recently been shown to be effective in rodent models of PTSD, including AC-5216/XBD173 and YL-IPA08 (Qiu et al., 2013).

Another neurosteroidogenic target is the pregnane xenobiotic receptor (PXR), a well-characterized, ubiquitous and promiscuous nuclear receptor important for metabolism and xenobiotic clearance in liver, kidney and intestine (Geick et al., 2001; Dussault and Forman, 2002; Francis et al., 2002; Kliewer et al., 2002). The recent discovery of PXR expression in brain has suggested a potential role for PXR in neural plasticity, as well. For example, PXR gene expression fluctuates across the estrous cycle in female rats and increases in the midbrain following mating, while knockdown of PXR expression in the ventral–tegmental area (VTA) reduces biosynthesis of ALLO in response to mating (reviewed in Frye, 2011; Frye et al., 2012, 2013). Inhibition of TSPO with the selective antagonist, PK11195, also reduces ALLO levels in midbrain, and reduces lordosis, effects reversed by ALLO administration. Together these data suggest that PXR may be upstream of TSPO (Frye et al., 2014).

The endocannabinoid system also has attracted attention as a steroidogenic target. The primary active ingredient of *Cannabis sativa*, Δ9-tetrahydrocannabinol (THC), increases pregnenolone synthesis in brain via activation of the type 1 cannabinoid receptor (CB1; Vallée et al., 2014). Other cannabinoid ligands thus are being studied for their potential as anxiety and PTSD therapies. There are interesting similarities between the cannabinoid system and ALLO in the regulation of emotion. Levels of ALLO and the endocannabinoid, anandamide (AEA) are decreased in models of stress-induced anxiety and depression (Matsumoto et al., 1999; Dong et al., 2001; Pibiri et al., 2008; Rademacher et al., 2008; Hill et al., 2009), and both ALLO and drugs that increase ALLO or AEA levels have similar effects on fear responses (Costanzi et al., 2003; Pibiri et al., 2008; Pinna et al., 2008; Lin et al., 2009).

The potential role of the endocannabinoid system in regulating emotional experience is further supported by the density of endocannabinoid receptors on glutamatergic neurons in emotion relevant areas such as the amygdala, hippocampus, and cortex (Slanina and Schweitzer, 2005; Katona, 2009). In addition, cannabinoids regulate intracellular peroxisome proliferator-activated receptors (PPARs), members of the nuclear hormone receptorsuperfamily (Forman et al., 1996; O’Sullivan, 2007; Pistis and Melis, 2010). The endocannabinoids, AEA and palmitoylethanolamide (PEA) are PPAR-α agonists, and PEA’s action at PPAR-α induces analgesia by enhancing ALLO biosynthesis (Sasso et al., 2012). A PEA-related increase in brain stem ALLO levels also potentiates pentobarbital hypnosis, an effect mimicked by PPAR-α agonists and prevented by ALLO biosynthetic enzyme blockers (Sasso et al., 2010, 2012). Also of note, PEA administration shows antidepressant effects equal to those of fluoxetine (Umathe et al., 2011; Yu et al., 2011) that activate ALLO biosynthesis (Pinna et al., 2003, 2006a).

The finding that activation of CB1 and PPAR-α receptors is capable of inducing ALLO biosynthesis, together with the pivotal role of ALLO in facilitating the action of GABA at GABA_A_ receptors, invites speculation about whether cannabinoid-related anxiolytic and anti-fear effects are due to the induction of corticolimbic ALLO biosynthesis. Cannabidiol, a non-sedating phytocannabinoid with a remarkably safe profile in humans, as well as other cannabinoids (Lin et al., 2006; Kobilo et al., 2007; Suzuki et al., 2008; Stern et al., 2012) have recently been shown to disrupt recent and older contextual fear memories by interfering with their reconsolidation. This effect of cannabidiol is long lasting and can be prevented by pharmacological antagonism of CB1 receptors (Stern et al., 2012). Interestingly then, the anti-fear effects of cannabidiol resulting in reconsolidation blockade were similar to the effects of midazolam, which like ALLO, activates GABA_A_ receptors (Stern et al., 2012).

The findings of the current study also support a role for GABA_A_ receptors in reconsolidation blockade and recovery from conditioned fear (Duvarci and Nader, 2004; Bustos et al., 2006). Administration of the ALLO-like compound, ganaxolone, during a critical time-limited window following exposure to conditioned contextual cues (Stern et al., 2012), markedly reduced the expression of fear in subsequent extinction trials and prevented the spontaneous recovery of fear (**Figure 5**). Given that PTSD is associated with benzodiazepine resistance, synaptic GABA_A_/benzodiazepine receptor complexes in humans with PTSD are decreased, and synaptic GABA_A_ receptors in the amygdala decrease after fear conditioning in rodents (Mou et al., 2011), it is tempting to speculate that blockade of reconsolidation may result from activation of extrasynaptic receptors, which are highly sensitive to neurosteroids (Belelli and Lambert, 2005). Furthermore, given that synaptic GABA_A_ receptors in the amygdala are restored after extinction of fear in rodents (Heldt and Ressler, 2007), it is possible that such restoration is a functional consequence of activation of extrasynaptic GABA_A_ receptors by GABAergic neurosteroids such as ALLO during extinction.

## CONCLUSION

Post-traumatic stress disorder appears to be a multifactorial disorder with several symptom clusters and involving neurochemical deficits that may vary among individuals with PTSD. Current treatments for PTSD are only efficacious in some patients or in some symptom clusters and not in others. Accumulated knowledge about the heterogeneous pathophysiology of PTSD thus suggests that treatments of the future should be “individually designed” rather than one-size fits all. In the case of PTSD patients who exhibit deficient ALLO biosynthesis and related deficits in GABAergic neurotransmission, ganaxolone administration may facilitate recovery. Perhaps then, future clinical trials of ganaxolone should be guided by pre-treatment ascertainment of ALLO levels and other relevant GABAergic system biomarkers as possible predictors of treatment efficacy.

## Conflict of Interest Statement

Marinus Pharmaceuticals provided ganaxolone to the DOD sponsored INTRuST Veterans Administration multi-site study of ganaxolone in PTSD, for which Christine Marx, MD, MA and Ann M. Rasmusson, MD are Co-Lead Investigators.

## Footnotes

1 http://clinicaltrials.gov

2 www.marinuspharma.com

3 www.cohort.com
